# Harnessing *Cannabis sativa* as a dual-use platform for biohydrogen production and pharmaceutical synthesis: a hypothesis and theory

**DOI:** 10.3389/fpls.2026.1833491

**Published:** 2026-05-20

**Authors:** Lei P. Wang, Babak Baban

**Affiliations:** 1 Georgia Institute of Cannabis Research, Medicinal Cannabis of Georgia LLC, Augusta, GA, United States; 2 Center for Excellence in Research, Scholarship and Innovation (CERSI), Dental College of Georgia, Augusta, Augusta University, Augusta, GA, United States

**Keywords:** biohydrogen, *Cannabis sativa*, circular economy, green hydrogen, photosynthesis, synthetic biology

## Abstract

*Cannabis sativa*, long established as a cornerstone of the pharmaceutical and industrial fiber markets, represents a radical and underexplored platform for renewable energy innovation. In this Hypothesis and Theory framework, we introduce a novel, patented (Provisional Patent No. 63916615) dual-use bio-refinery paradigm. This model harnesses engineered cannabis photosynthesis to drive green hydrogen production without compromising its established value as a high-yield medicinal crop. By strategically redirecting photosynthetic electron flow toward oxygen-protected hydrogenase activity, it is possible to generate molecular hydrogen at commercially relevant scales while maintaining plant viability. Unique to this model is the ability to leverage over $10 billion in existing controlled-environment agriculture (CEA) infrastructure, bypassing the capital-intensive barriers that have hindered traditional algal biohydrogen systems. We outline a tripartite circular economy strategy that integrates hydrogen capture during the vegetative phase with the subsequent harvest of therapeutic cannabinoids and industrial biomass. This convergence of synthetic biology, clean energy, and biomedicine positions cannabis as a uniquely versatile multipurpose crop capable of fueling both the pharmaceutical industry and the global transition to a sustainable hydrogen economy.

## Graphical Abstract

**Figure:**
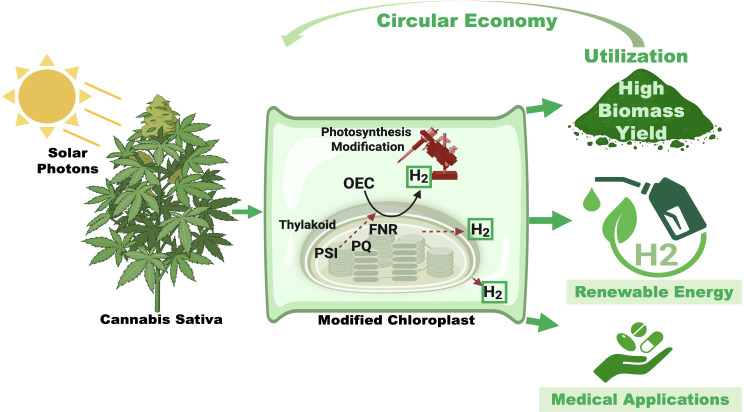
A visual overview of the circular economy model, showing solar photons driving modified chloroplasts to produce high biomass, renewable *H_2_*, and medical applications.

## Introduction

The global transition toward a net-zero carbon economy necessitates the development of scalable, carbon-negative energy sources (Ali et al., 2024). While hydrogen (*H_2_*) is a premier clean energy carrier, biological production methods have traditionally struggled with economic viability due to low feedstock density and high infrastructure costs (Chandrasekhar et al., 2015; Weixian et al., 2023). We propose that Cannabis sativa, a crop already optimized for high-density biomass and metabolic output, serves as the ideal biological “factory” to overcome these hurdles.

## Rationale and advantages of cannabis as a hydrogen crop

The rationale for advancing *Cannabis sativa* as a biohydrogen crop rests on its unique combination of agronomic, societal, and biotechnological advantages (Hussain et al., 2021; Brar et al., 2022). Unlike algae, where biomass is largely consumed during production, cannabis offers a multipurpose lifecycle that ensures value generation beyond energy capture (Brar et al., 2022; Ahsan et al., 2025). As detailed in **Table 1**, the cannabis biorefinery model offers significantly higher Net Present Value (NPV) and a faster Return on Investment (ROI) compared to traditional green algae or cyanobacteria systems, primarily due to the secondary market for high-value metabolites.

**Table 1:** Comparative analysis of biohydrogen feedstocks.

Parameter	Cannabis sativa	Green algae	Cyanobacteria	Cannabis advantage
H2 Yield	0.5-2 mmol/g/h	2-5 mmol/g/h	0.5-3 mmol/g/h	Lower but offset by multi-product
Biomass	20-30 ton/ha/yr	10-50 ton/ha/yr	5-20 ton/ha/yr	2-3x higher
Product Value	Very High	Low	Low	$10K-50K/ha cannabinoids
Land Use	High	Very High	High	Existing infrastructure
Water	Moderate-High	High	Moderate	Comparable
Infrastructure	High (farms exist)	Low (need reactors)	Low (need facilities)	$10B+ industry
Food Competition	None	None	None	Ethical advantage
Regulatory	High (GMO+cannabis)	Moderate (GMO)	Low-Moderate	Complex but doable
Scalability	High (field)	Moderate (reactors)	Moderate (ponds)	Agricultural advantage
O2 Sensitivity	High	High	Moderate	All face challenge
Tech Readiness	TRL 2-3	TRL 4-6	TRL 4-5	Catching up
Economic Model	Multi-revenue	Single revenue	Single revenue	Risk diversification
Market	Energy + Medical	Energy	Energy	Bridges two markets
NPV (20-year)	$500K-2M/ha	$100K-300K/ha	$50K-200K/ha	3-5x higher
Break-Even	3-5 years	7-10 years	8-12 years	Faster ROI

*Estimates based on theoretical calculations.

The superiority of this platform is further evidenced by several key factors:

- Biomass & Photosynthesis: Its rapid growth and dense foliage contribute to exceptional biomass yields and elevated photosynthetic capacity.
- Non-Food Status: As a non-food crop, it avoids the ethical “food vs. fuel” concerns associated with diverting edible crops like corn for ethanol.
- Pharma-Energy Convergence: Its leaves and flowers provide therapeutic and nutraceutical products (CBD, terpenes), while stalks generate industrial-grade biomass.
- Infrastructure Adaptability: Legalization trends have driven major advances in Controlled-Environment Agriculture (CEA). This established infrastructure, valued at over $10 billion, is readily adaptable for energy farming, significantly lowering the barrier to entry for green hydrogen production.

*Cannabis sativa* has undergone a dramatic transformation in its industrial status over the past decade. As of 2025, the global industrial hemp market is valued at approximately $7.5 billion and is projected to exceed $27.7 billion by 2033, driven by demand across textiles, nutraceuticals, construction materials, and cosmetics (Grand View Research, 2025). In the United States, the 2018 Farm Bill federally legalized hemp cultivation, triggering rapid expansion of CEA infrastructure specifically designed for cannabis production, now estimated to exceed $10 billion in capital investment. Internationally, the European Union, Canada, and Australia have established regulatory frameworks supporting both medicinal and industrial hemp. This existing and growing industrial infrastructure represents a critical enabling factor for our proposed dual-use biorefinery model, as the capital-intensive barrier to entry for green hydrogen production is substantially reduced when built upon an already-established cannabis cultivation ecosystem.

## The dual-use biorefinery paradigm (the hypothesis)

We hypothesize that *C. sativa* can be biotechnologically steered to function as a dual-output system by integrating hydrogenase-expressing synthetic pathways directly into the chloroplast genome. This approach enables the capture of molecular *H_2_* during the plant’s intense vegetative growth phase, without compromising the downstream production of high-value cannabinoids and industrial biomass. Unlike conventional biohydrogen systems that sacrifice biomass for energy, this dual-use paradigm preserves, and indeed leverages, the full metabolic output of the plant across its lifecycle.

## Synthetic biology and chloroplast modification

The primary challenge in biological *H_2_* production is the sensitivity of hydrogenase enzymes to the oxygen (*O_2_*) produced during photosynthesis (Cha et al., 2025). To overcome this, our model proposes the engineering of an oxygen-protected niche within the chloroplast. At the molecular level, our proposed engineering strategy involves three key interventions. First, heterologous expression of an oxygen-tolerant [FeFe]-hydrogenase, such as those characterized in Clostridium acetobutylicum or engineered variants from Chlamydomonas reinhardtii, into the cannabis chloroplast genome via plastid transformation (Demuez et al., 2007; Wegelius et al., 2018). Second, targeted downregulation of competing electron sinks, specifically the ferredoxin-NADP+ reductase (FNR) pathway, to redirect photosynthetic electron flow from carbon fixation toward proton reduction. Third, co-expression of oxygen-scavenging enzymes (e.g., glucose oxidase or leghemoglobin) within the chloroplast stroma to create and maintain the low-O_2_ microenvironment essential for sustained hydrogenase activity (Demuez et al., 2007; Appel et al., 2022). Together, these modifications create a metabolically partitioned chloroplast capable of simultaneous carbon fixation and hydrogen evolution.

**Figure 1:**
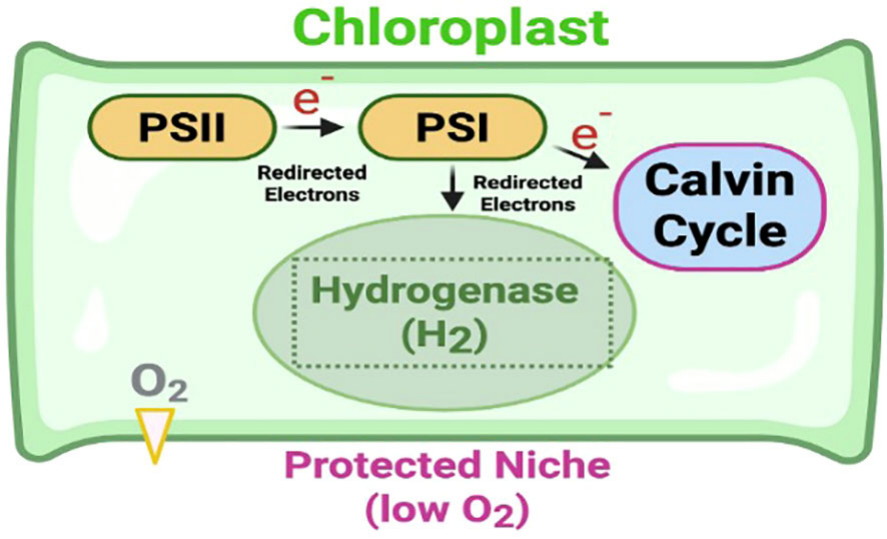
Schematic of synthetic chloroplast engineering for photobiological hydrogen production in *Cannabis sativa*. The proposed model illustrates the redirection of the photosynthetic electron transport chain to drive hydrogen evolution. Light energy harvested by Photosystem II (PSII) and Photosystem I (PSI) generates high-energy electrons that are traditionally used for *CO_2_* fixation via the Calvin Cycle. In this engineered paradigm, a fraction of these electrons is intercepted and funneled toward a heterologously expressed hydrogenase (H2ase) enzyme. To prevent the characteristic inhibition of H2ase by photosynthetic *O_2_*, the enzyme is sequestered within a specialized, oxygen-protected niche (shaded area) within the chloroplast stroma. This spatial or temporal separation allows for the continuous reduction of protons (*2H^+^*) into molecular *H_2_* without interrupting the primary metabolic processes required for plant growth and biomass accumulation.

As illustrated in **Figure 1**, the proposed synthetic pathway involves redirecting the photosynthetic electron flow. Under specific physiological conditions, electrons generated from Photosystem II (PSII) and Photosystem I (PSI) are intercepted and funneled toward a heterologously expressed hydrogenase (H2ase). This enzyme, localized within a protected micro-compartment, facilitates the reduction of protons into molecular *H_2_* without compromising the primary carbon fixation required for biomass accumulation (Wu et al., 2010; Wegelius et al., 2018).

## The tripartite sustainability cycle

The economic viability of our proposed dual-use paradigm relies on a staggered, multi-phase harvest model. Unlike traditional bioenergy crops that are destroyed during energy extraction, *C. sativa* facilitates a sequential recovery of value.

**Figure 2:**
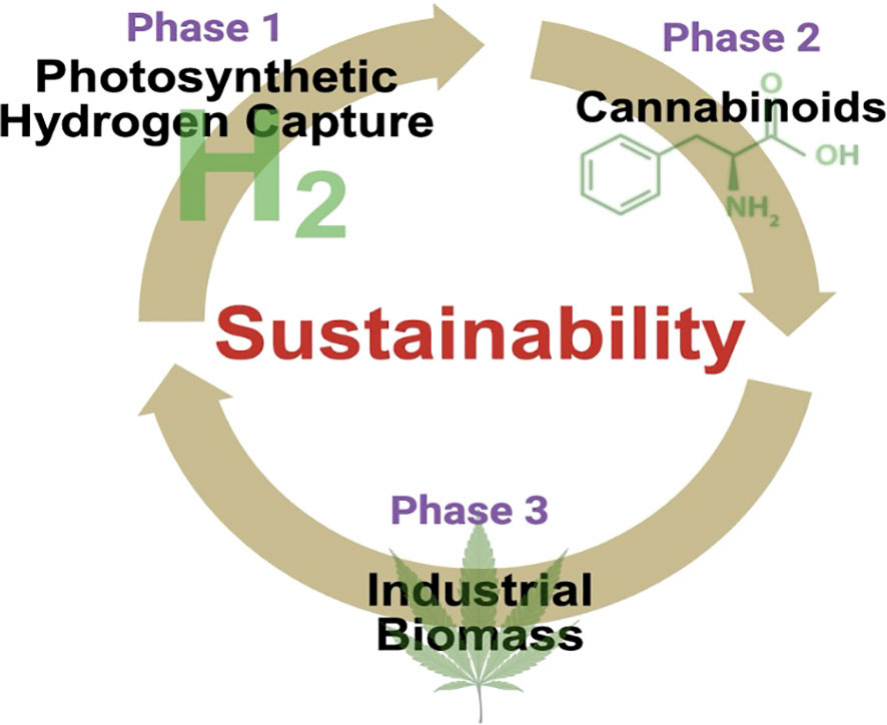
The tripartite circular economy strategy for dual-use *Cannabis sativa* biorefining. This schematic illustrates the sequential value-capture model of the proposed hypothesis. Phase I (Capture): Illustrates the capture of renewable green *H_2_* generated during the vegetative growth phase via modified photosynthetic pathways. Phase II (Synthesis): Highlights the subsequent maturation of the plant, where metabolic precursors are diverted into the synthesis of pharmaceutical-grade cannabinoids (CBD/THC) and terpenes. Phase III (Recovery): Demonstrates the final utilization of residual lignocellulosic biomass for industrial applications (e.g., textiles, construction materials) or conversion into biochar for soil amendment and carbon sequestration. This integrated approach ensures that the high market value of pharmaceutical outputs effectively subsidizes the cost of green energy production.

As depicted in **Figure 2**, the Tripartite Sustainability Cycle begins with Phase I: Photosynthetic *H_2_* Capture. During the peak vegetative growth stage, gaseous *H_2_* is harvested within closed-loop CEA systems. Following this, the plant enters Phase II: Cannabinoid Synthesis, where the metabolic energy is redirected toward the production of high-value secondary metabolites (e.g., CBD and terpenes) in the glandular trichomes. Finally, Phase III: Industrial Biomass Recovery involves the processing of the remaining stalks and shives into industrial fiber or carbon-sequestering biochar, ensuring a zero-waste, circular economy.

Beyond hydrogen and cannabinoids, *Cannabis sativa* offers a remarkably diverse portfolio of industrial outputs that strengthen its circular economy value proposition. Hemp fiber derived from the stalks is among the strongest natural fibers known, with applications in textiles, construction composites, and biodegradable packaging (Enarevba and Haapala, 2024; Shelly et al., 2025). Hemp-derived biochar, produced from post-harvest lignocellulosic residues via pyrolysis, serves as a long-term carbon sequestration agent and soil amendment, improving water retention and microbial diversity in agricultural settings (Puglia et al., 2023). Hemp seed oil, rich in omega-3 and omega-6 fatty acids, has applications in nutraceuticals, cosmetics, and bioplastics (Brar et al., 2022). This multi-stream valorization model ensures that virtually no biomass fraction is wasted, positioning cannabis as one of the most versatile and economically resilient crops available for circular bioeconomy integration.

## Strategic implementation and economic outlook

The feasibility of our dual-use paradigm depends on the efficiency of photosynthetic *H_2_* evolution. While wild-type *C. sativa* exhibits baseline levels of *H_2_* production under certain anaerobic conditions, it is insufficient for industrial energy capture.

**Figure 3:**
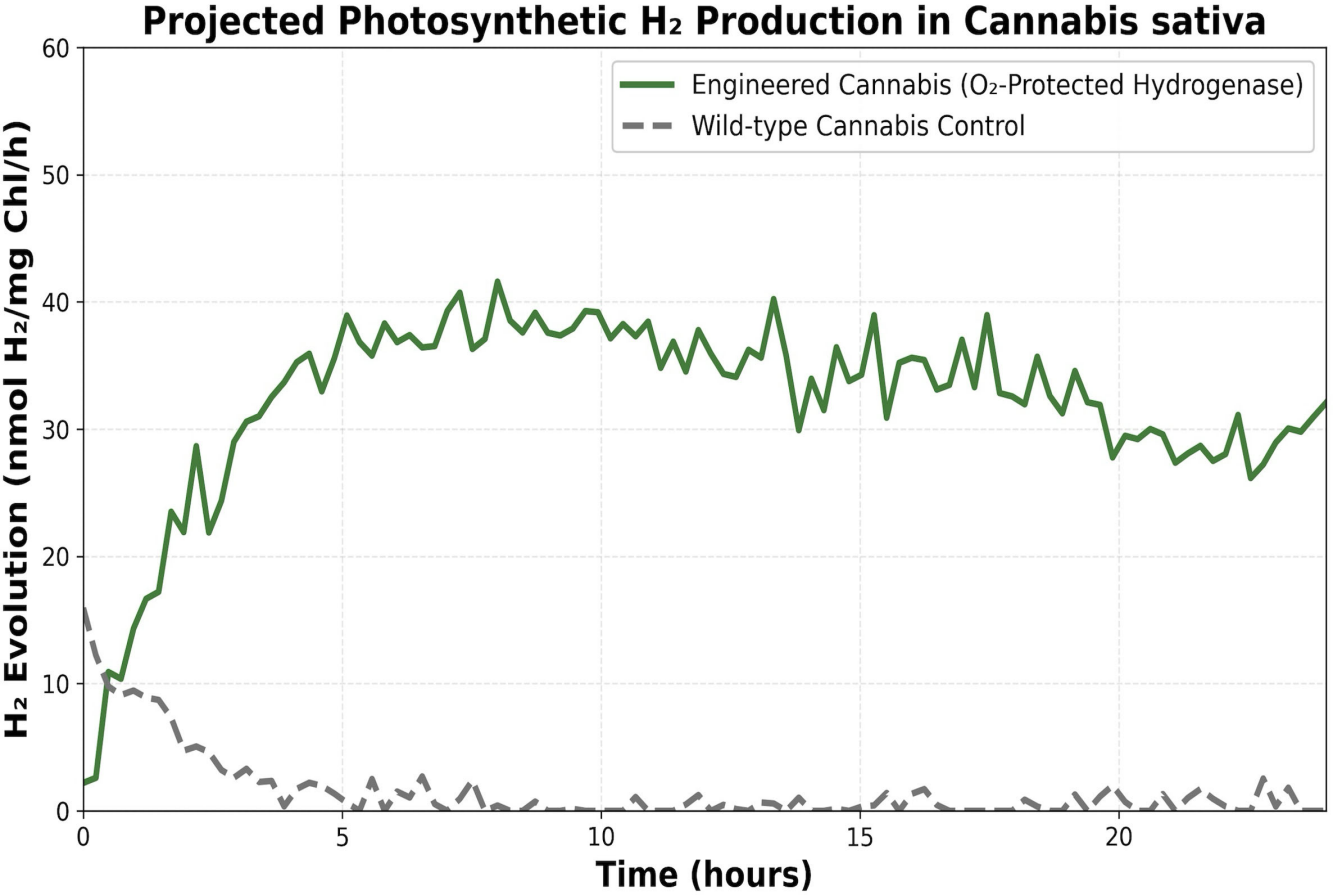
Projected photosynthetic *H_2_* production in engineered vs. wild-type *Cannabis sativa*. The graph illustrates the hypothesized kinetics of molecular *H_2_* evolution over time. Wild-Type (WT): Represents the baseline, transient *H_2_*production observed under natural physiological conditions. Engineered (Modified): Demonstrates the sustained and significantly elevated *H_2_* evolution rates achieved through the integration of oxygen-protected hydrogenase and redirected photosynthetic electron transport. The vertical axis represents the rate of hydrogen production normalized to chlorophyll content (*nmol H_2_/mg Chl/h*), while the horizontal axis depicts the duration of the vegetative growth phase. The shaded area represents the “Capture Window” for renewable energy, occurring prior to the metabolic shift toward cannabinoid synthesis (Phase II).

As projected in **Figure 3**, our synthetic biology approach aims to achieve sustained, high-level *H_2_* evolution. By optimizing the electron flux and protecting the hydrogenase enzyme (as detailed in **Figure 1**), the engineered strains are expected to maintain an elevated production rate (*nmol H_2_/mg Chl/h*) throughout the vegetative growth phase. This sustained output, compared to the transient and low-level production of wild-type controls, provides the necessary volume for capture within Controlled-Environment Agriculture (CEA) facilities. This elevated yield, combined with the $10 billion existing infrastructure, positions the *Cannabis* biorefinery as a commercially viable source of green *H_2_*.

## Conclusion

*Cannabis sativa* stands at the intersection of the most disruptive shifts in modern industry: the legalization of medicinal biotechnologies and the urgent need for carbon-negative energy transition. By adopting this patented dual-use framework, we can transform one of the world’s most valuable crops into an engine for a sustainable, hydrogen-powered future.

Several significant challenges must be acknowledged in the translation of this hypothesis to practice. First, the genetic transformation of *Cannabis sativa* chloroplasts remains technically demanding, as stable plastid transformation protocols for cannabis are less established than for model organisms such as tobacco (Wegelius et al., 2018; Narra et al., 2025). Second, the metabolic burden imposed by heterologous hydrogenase expression may compete with cannabinoid biosynthesis, requiring careful regulatory tuning of gene expression across growth phases. Third, the scalability of *H_2_* capture within CEA systems introduces engineering challenges related to gas-tight enclosures, hydrogen safety, and capture efficiency. Fourth, the regulatory landscape governing genetically modified cannabis varies considerably across jurisdictions, which may limit near-term deployment in certain markets (Hussain et al., 2021). To overcome these challenges, we propose a phased development roadmap: beginning with proof-of-concept studies in model plant systems (e.g., tobacco), followed by transient expression studies in cannabis, and ultimately progressing to stable chloroplast transformation with regulatory engagement. Collaborative frameworks between synthetic biologists, agricultural engineers, and regulatory bodies will be essential to advance this platform toward commercial viability.

## Data Availability

The original contributions presented in the study are included in the article/supplementary material. Further inquiries can be directed to the corresponding author.
